# The Hospice as a Learning Environment: A Follow-Up Study with a Palliative Care Team

**DOI:** 10.3390/ijerph17207460

**Published:** 2020-10-14

**Authors:** Ines Testoni, Vito Fabio Sblano, Lorenza Palazzo, Sara Pompele, Michael Alexander Wieser

**Affiliations:** 1 Department of Philosophy, Sociology, Pedagogy and Applied Psychology (FISPPA), University of Padova, 35122 Padova, Italy; ines.testoni@unipd.it (I.T.); sblanofabio@gmail.com (V.F.S.); lorenza.palazzo92@gmail.com (L.P.); sara.pompele93@gmail.com (S.P.); 2 Emili Sagol Creative Arts Therapies Research Center, University of Haifa, Haifa 3498838, Israel; 3 Department of Psychology, University of Klagenfurt, Klagenfurt 9020, Austria

**Keywords:** hospice, death education, palliative care team, community

## Abstract

In Western society, the topic of death has been removed from everyday life and replaced with medical language. Such censorship does not reduce individuals’ fear of death, but rather limits their ability to elaborate their experiences of death, thus generating negative effects. The objective of this follow-up qualitative study was to detect how and if death education can help to improve individuals’ relationship with death and enhance care environments like hospices. Semi-structured interviews were conducted with palliative care professionals and teachers who had taken part in a death education initiative three years earlier. The results confirmed the initiative’s positive effect on both palliative care professionals and teachers. The participants reported that the education initiative helped them to positively modify their perspective on death, end-of-life care, and their own relationship to life, as well as their perception of community attitudes towards the hospice, which seemed to become less discriminatory. This study confirmed that school education initiatives can usefully create continuity between hospices and local communities. This project provided an educational space wherein it was possible for participants to elaborate their experiences in relation to death and to re-evaluate and appreciate hospices.

## 1. Introduction

The theme of death in Western society has been removed from everyday life and replaced with specialised medical language. This may be related to people’s innate tendency to avoid anything that reminds them of their finitude [1]. As asserted by terror management theory (TMT), awareness of death is inevitable, and such awareness may occur at any moment for uncontrollable reasons and generate a feeling of latent and constant terror. According to this perspective, this reaction is natural because the terror of death can become paralysing, thus preventing the normal flow of everyday life [2]. Indeed, consciousness of one’s own mortality, or “mortality salience”, can paralyse individuals to the extent that they are unable to react effectively to environmental hazards [2]. Two psychological strategies are employed to defend against the terror of death: “proximal defences”, which enable people to remove the thought of death from conscious attention, and “distal defences”, which reduce the accessibility of the thought, thereby reducing the likelihood of death-related thoughts returning to focal attention. Distal defences often involve existential reflection on the meaning of finitude and the construction of cultural apparatuses that allow individuals to give meaning to their lives and actions. Religions, for example, are very important in this area because they make it possible to envisage individual existence beyond earthly life and beyond death [3,4]. However, these systems are fragile because they are constantly being questioned by common and rational thought [5,6,7,8]. The resulting uncertainty leads to the widespread use of unconscious defences. One of the clearest examples of this type of denial can be seen in the stigmatisation of those who work in funeral homes or morgues [9,10,11], as well as those who work in hospices [12,13,14,15,16,17,18]. The avoidance of conversations or individuals associated with death may constitute a proximal defence. Indeed, one hypothesis derived from TMT, known as death-thought accessibility (DTA), states that if a psychological structure provides protection from thoughts of death, when it weakens, it should momentarily bring death thoughts closer to consciousness [19]. This means that the weakening of cultural (distal) defence structures inevitably increases death-thought accessibility. According to the DTA perspective, stigmatisation can originate from people’s failure to activate distal defences due to the lack of sufficiently strong cultural structures.

Furthermore, this social denial contributes to a reduced awareness of finitude among young people who are surrounded by adults who are incapable of addressing the topic in their daily lives [20,21]. This type of censorship restricts the possibility of developing a symbolic language to elaborate the experiences that accompany the terror of death [22]. To mitigate this problem, school environments could provide educational programs that allow children to talk about death and to describe mourning situations that they may be experiencing. Death education courses are designed to offer precisely this type of training, both in schools and in the community. In schools they allow children and adolescents to deal with death regardless of their pre-existing familiarity or experience with this topic [23,24].

Death education initiatives create a space in which to explore the various ways of conceptualising death, which enables people to address their own death anxiety by eliminating the fantastic, magical and persecutory connotations of death (often acquired in childhood) and leads to a greater understanding of personal value systems and a more mature interpretation of death [25,26,27,28,29,30,31]. The main purpose of death education is to integrate death into life, and this can be implemented as a preventive intervention [32,33,34]. Death education in community settings, such as schools, also allows young people to get to know each other, increases peer support, teaches children listening and empathic skills, and encourages them to withhold judgement [23,34,35]. It can be implemented in many ways including lectures on death and dying and more informal activities such as focus groups, photovoice, psychodrama, bibliodrama, etc. [1,20,28,36,37,38].

In 2016, death education was implemented in three schools in a small town in Southern Italy in response to a teenage student’s suicide. High schools in the area decided to address this bereavement and the wider theme of death with students [38]. The project was discussed and developed by death education experts and some of the teachers who had personally dealt with the consequences of the student’s suicide. The fundamental objective was to break down the barriers around the topic of death in order to discuss death freely, thereby giving free rein to feelings, fears and anxieties, while also making it possible to answer students’ questions concerning the meaning of life using philosophical, religious and artistic content. For this reason, the organisers decided to involve the local hospice.

Meetings between the hospice operators, experts and teachers created a very tight-knit team, and the project was implemented with lessons at the schools and in the hospice [38]. Before the start of the death education program, meetings were held with students to see if they would agree to participate. Following these discussions, 138 students (males, *n* = 63; females, *n* = 75) from three high schools enrolled in the death education initiative. The initiative drew on a model based on previous death education experiences [39] and aimed to provoke reflection on the differences and relationships between “my own death” and “others’ death”. The program was divided into two parts, in the first part, formal lessons were given on philosophical approaches to death and spirituality and on meditation (in Western and Eastern traditions) by psychologists specialised in death education and an expert in religious education. Additionally, informal lessons were given on meditation, psychodrama and moviemaking to enable students to reflect on the relationship between mortality and transcendence and on the meaning of suicide. During the informal lessons, students also shared their feelings and thoughts in small-group discussions, participated in psychodrama sessions where the techniques of soliloquy, doubling and role reversal were used, and at the end, they produced a movie about this experience.

In the second part, the students visited a local hospice where they had the opportunity to interact with doctors, nurses, religious officials and psychologists to discuss death and dying, how patients were supported by the palliative care team and how their families handled their grief. This part of the death education initiative was designed to involve students in the discourse of death, and to grant them access to people who routinely deal with death and who could provide a positive example of healthy emotional management thanks to their ability to comprehend and experience pain—both their own and that of others. It also aspired to demonstrate how people handle situations of illness with a poor prognosis and how they can be helped by support from the community. We hoped to make it clear that community members can help the professionals and the families of those who are dying by changing their understanding of the hospice, which was too often seen as a “place of death” inhabited by “angels who bring death”. The model of palliative care was explained to students, with particular focus on how staff members attend to those who suffer, honour the value of life and promote its quality right up to the last moment [40]. This step encouraged the students to reflect on the gravity of death and dying while also acknowledging the generosity of those who work competently and compassionately to enhance patients’ quality of life. In this way, it was possible to highlight that life is a precious gift that must be safeguarded while also affirming that death should not be denied (or prolonged or accelerated). The discussion component focused on how the death of a loved one prompted relatives and friends to consider huge existential questions.

Two years after the positive conclusion of the project, some of the school teachers proposed that we redo the program. Before starting all the planning work again, however, we thought it was necessary to explore teachers’ and hospice professionals’ perspectives on the initiative’s outcomes in order to assess the value of a second iteration. As such, the present study investigates the effects of the experience on the program participants (teachers/hospice staff) who had taken part in the aforementioned death education initiative three years previously.

## 2. Materials and Methods

This research followed the grounded theory perspective [41] as it explored a field that is not often addressed and regularly censored, as the founders of grounded theory themselves noted when they began to study how people deal with death and dying [42]. The main objective was to detect (1) whether death education had a positive impact on teachers and hospice professionals, and (2) whether they experienced or observed a positive change with respect to their relationship with representations of death. Furthermore, we wanted to know if the initiative had changed the relationship between the community and the local hospice, from the point of view of both the teachers and the hospice professionals. Finally, the research analysed how these participants interpreted the initiative’s effect on students’ representations of death, as well as their perspective on its effect on the community’s attitudes towards the hospice.

The interviews were carried out in places chosen by the participants. Each interview lasted about 60 min and was recorded and transcribed verbatim in Italian. The texts thus obtained were then subjected to thematic analysis using Atas.ti software developed by Thomas Muhr at the Technical University of Berlin (1989–1992) and is produced by ATLAS.ti Scientific Software Development GmbH [43,44], which focused on identifying recurring meanings and key concepts [45,46]. This thematic analysis shed light on patterns in the dataset [29], and allowed us to develop a conceptual framework. We ensured the credibility of our methodology by following the consolidated criteria for reporting qualitative research (CORE-Q) checklist [47] and thematic analysis [45,46]. Each process was based on the researchers’ familiarity with the previous education experience and with the categories that arose as the analysis progressed [36,39]. The analysis occurred in six main phases: preparatory organisation, generating categories or themes, coding data, testing emerging understanding, searching for alternative explanations, and writing up the report [43,48,49].

Lastly, in order to check the reliability of our results, we sent a write-up of our analysis to the participants at the end of the process (after six months), asking them to give us their opinions or further suggestions. In particular, we asked them whether they believed that the texts were consistent with what had happened and what they had experienced.

The study involved 20 individuals who had taken part in the death education project three years earlier: 11 members (males, *n* = 5; females, *n* = 6) of a local hospice aged between 29 and 50 years (µ = 44; σ = 5.98) and nine school teachers aged between 29 and 63 years (µ = 48.1; σ = 9.18). The hospice staff comprised two psychologists, three doctors, four nurses, a family liaison officer, and a spiritual guide. Among the hospice staff, 70% of individuals were university graduates, eight individuals were married/cohabiting, and nine individuals had children. All the interviewees were Italian citizens.

The teachers who took part in the education initiative were staff members of local schools in the town where the original study took place. In the study group, 90% of the participants were teachers who taught humanities classes (religion, history and Italian), and their ages ranged between 31 and 63 years (µ = 53.11; σ = 10.20). Seven were married with children and two were unmarried. All the teachers were university graduates and possessed Italian citizenship (Table 1).

As the students who had participated in the original education initiative had finished their studies and could not be traced due to their anonymity in the original project, they could not be included in the present research.

The study was conducted in accordance with the Declaration of Helsinki. The study was approved by the Ethics Committee for Psychological Research of the University of Padova, Italy (No. B9A50488FF46457ADE1F0F9A4FC5760E). Each participant was asked to sign a written informed consent form authorizing their participation, the data treatment, and the audio-recordings of the interviews.

## 3. Results

Based on the participants’ responses, three main thematic areas emerged: (1) changes following the death education initiative, (2) the usefulness of the death education initiative for elaborating the community’s traumatic grief, and (3) motivations to reintroduce the death education initiative.

The names of the participants have been changed to preserve their anonymity.

### 3.1. Thematic Area 1: Changes Following the Death Education Initiative

Some participants noted that the theme of death was not novel because the traumatic grief surrounding a student’s suicide had previously forced the community to ask questions about the issue of youth suicide. Some teachers reported that there were feelings of disbelief linked to this traumatic episode, and said that the community acted as though the boy was missing rather than dead. Members of the community struggled to convince themselves that it was a definitive event; the common feeling was that the boy would come back as if he had only gone away momentarily.

The experience of death education, and in particular, the visit to the hospice, made it possible to deal with this disbelief and denial and to reflect on its meaning. This in turn enabled an exploration of ontological representations of death, as explained by Andrea, one of the hospice nurses:

“The death education course allowed me, in the first place, to examine the ontological representations I had concerning death and, while reflecting upon them, to modify the most distressing ones. Initially, I saw death as something to avoid, something I preferred not to think about, that I preferred to cast aside, and now, instead, I am able to face the idea of death in a calmer way, I am able to speak about it, to mention it; I am able to accept death as, indeed, the conclusion of a path that is part of life. This allows me to act as a support for the patients’ relatives in the hospice and also as a support for my own relatives and friends.”

For most of the hospice staff, understanding the meaning of the term “passage” and being able to question it was important. For some people, this meant being able to reconsider their idea of death, seeing it not as the absolute end, but rather as a passage. This also helped to change their view of life, conceiving it as a succession of experiences that are preserved after death. Some of the participants expressed this renewed vision of life as a greater appreciation for the little things in life, such as waking up in the morning and going to sleep at night, or as a process of learning to turn the fear of death into a desire to live. Some teachers came to understand and accept that thoughts about death do not have to undermine life, as they can actually allow people to live more consciously and more fully by teaching them to grasp the nuances of life that were previously taken for granted.

The teachers also perceived a change in students’ vision of life, relaying statements that students had shared with them about understanding the importance of each moment in life and being present with people who suffer, even in silence. According to the teachers, the students grew up after this experience, and the stories that they exchanged about their experiences of loss made them feel closer to each other and part of the “great mystery of life”.

There was also a shift in how the community viewed the hospice and its staff after the death education project. Prior to the project, the hospice and hospice staff were surrounded by an aura of mystery and fear because of the community’s prejudice (i.e., social censorship). As Giulio affirmed:

“The general fear was of entering a sort of “factory of death”. When I proposed to visit the hospice, the students were full of prejudices that certainly came from what we, as adults, communicate to them, from the way we talk about the hospice in our society.”

**Figure 1:**
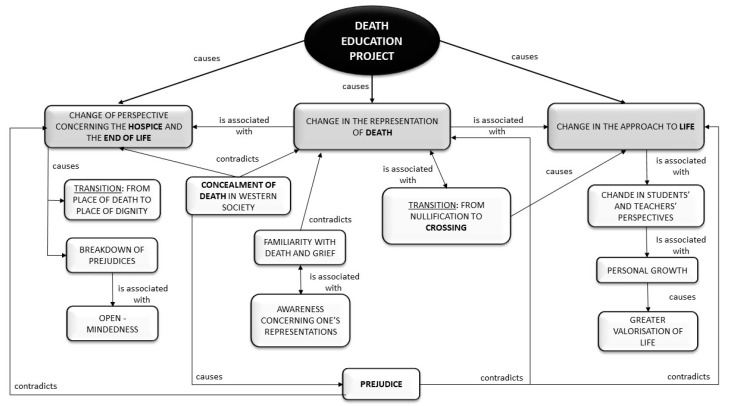
Changes following the death education initiative.

All of the teachers stressed the usefulness of the intervention for overcoming these prejudices, shedding the belief that the hospice was a place of sadness and death and coming to consider it, instead, as a place full of dignity. Before this experience, some of the teachers knew very little about the difficulties that are common at the end of life or the kind of work that hospice professionals do, and they were very impressed by the hospice staff’s humanity. In the same way, the students also had a change of heart after this experience, and they shared these new perspectives at home and with their friends. They told the teachers that they felt less fear and anguish about death and that they were able to look inside themselves and better understand their emotions and those of their classmates (Figure 1).

### 3.2. Thematic Area 2: Usefulness of Death Education for Elaborating the Community’s Traumatic Grief

Almost all the teachers and hospice staff spoke positively about the initiative’s effects when it came to elaborating traumatic grief. Valeria, a psychologist at the hospice, affirmed:

“There is a huge difference between being silent, as often happens after a suicide, and being able to talk about it, considering death as something natural. In the latter case, the elaboration of grief helps people to draw on the resources they need to face the situation and the distress without isolating themselves … on the contrary, talking about grief at the community level offers support to all.”

Fabio, a doctor, declared:

“Talking about death can make us reflect upon the fact that death is part of life and that it is therefore not something obscene but rather something that is natural. It is important to intervene with a philosophical reflection concerning life, considering it as a good that is not endless, but, on the contrary, available for a limited amount of time.”

Hospice workers reported that they are willing to take further steps of this kind, and that it is their intention to pursue networking between end-of-life workers and the community to promote a cultural change in representations of death. One nurse, Lucio, said:

“Death education is useful because it means going back to our origins; in the past, indeed, when there wasn’t a very sophisticated health network, these efforts to accompany people facing death were conducted at home, and therefore there was much more solidarity. A communitarian path would help us rediscover what we already have inside of us, that is, the importance of accompanying a person right until his/her last instant of life. Thanks to these death education courses, there is a more welcoming atmosphere in the hospice, and the sense of responsibility towards the patients and their families has increased because we now operate in a cultural environment that needs to seriously deepen its engagement with the themes of death.”

**Figure 2:**
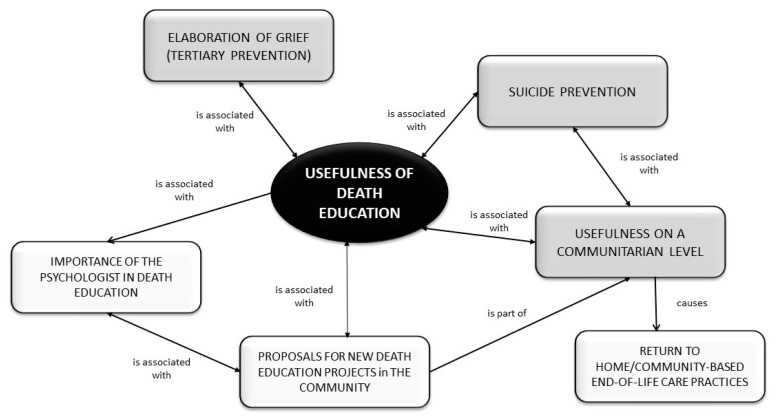
Usefulness of death education.

Many of the teachers also expressed very positive views regarding this aspect of the death education initiative. Some reported positive feedback from the students, saying that they have been able to talk about the loss of their friend and thus to move on with their lives in a more peaceful and serene way. In addition, some teachers claimed that the initiative helped them and the children to accept that the path of life, which ends with death, is made of both satisfactory steps and steps full of suffering that cannot be avoided. They believe that community support and sharing can help to make people feel less lonely by involving them in meaningful relationships, which is an important factor that protects against suicide. The teachers’ testimonies on the usefulness of death education at the community level appeared to be just as positive as those of the hospice staff. In some cases, they favoured a more proactive approach, with some putting forward innovative proposals to facilitate death education in schools, parishes and city squares. They stated that this would be especially useful because it could extend psychological help to patients’ relatives, and also because it would promote human growth: everyone could become aware of the fact that every one of us will eventually live the experience of death (Figure 2).

### 3.3. Thematic Area 3: Motivations to Reintroduce the Death Education Initiative

Hospice workers reported that they would be willing to redo this death education course and welcome students into the hospice again because they believe in its educational, pedagogical and formative potential to foster understanding and acceptance of separation, loss and illness–challenges that are close to everyone. According to some of them, this initiative also helped the professionals themselves to re-examine their knowledge and skills and to question these with the students.

From the teachers’ point of view, the initiative was significant for them because it helped them to relate to each other and to their students, thus breaking down barriers so that they were able to become close enough to share their emotions. Some teachers also found it useful because it equipped them with tools that they can use to talk about these themes at home with their own children, who, they fear, may otherwise be unable to deal with the thought of illness or death.

**Figure 3:**
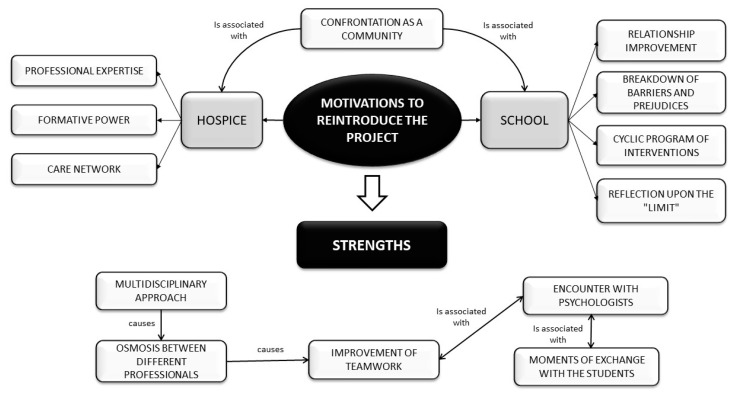
Motivation to reintroduce the project.

Both the teachers and the hospice staff believed that current students would also welcome this course. Teachers argued that the course should be implemented every year as part of the school curriculum, as it is important for high-school-aged students to reflect on these topics. They also said that the whole community should know about this curriculum, as they would be affected as a result. According to teachers, future iterations of the initiative should maintain the original multidisciplinary approach and the opportunity to meet different people working at different levels of end-of-life care. Moreover, the initiative should preserve a space for students to open up amongst themselves with regard to their experiences, fears and anxieties about death. Some teachers reported that they were impressed that students were having these conversations with their classmates in small groups, as before they confided only with the teachers on these issues, and only outside the classroom. This also allowed them to resolve some critical issues in class, such as quarrels and discussions, and to build relationships based on empathy. The teachers affirmed that the visit to the hospice was a fundamental step because it allowed the students to reevaluate a place that their community had encouraged them to avoid. Then, thanks to the students, this new orientation can spread within families and be transmitted “like osmosis” throughout the community (Figure 3).

In the final phase of the research, we submitted the results of our analysis to the participants alongside a final written report presented by the interviewer, and asked them whether they confirmed the findings. The answers were all positive, though some of the respondents suggested additional considerations. For example, the priest, Luigi, emphasised that “The tests are going great. I confirm that the project has paved the way for a new understanding of palliative care and spiritual accompaniment in the hospice team’s work”. Among the nurses, Maria affirmed:

“Yes, all well and good, but I would like to stress more that here in the hospice, we were all amazed at the maturity shown by the students. They showed great depth of thought in their discussions with us, despite their young age, and this is important because it means that they really think about death even though no one seriously talks to them about it, and this course permitted them to reflect on this issue in a more profound way.”

One teacher, Giovanni, said:

“Yes, I absolutely agree, but I want to make some additions. It is very important to affirm that all the schools should implement death education courses, inserting them into the educational system in a structural and interdisciplinary way. In fact, I do not believe that only spiritual dimensions can help to manage death. Indeed, there are spiritual texts of great importance, but also theatrical, musical and pictorial works. Art also helps to manage anguish. We all need good and beautiful readings that enrich our language and our ability to attach words to our experiences of loss and mortality.”

Another teacher, Francesco, wanted to add:

“I agree with everything, but now that I have the chance, I feel I still have something to express. The project has highlighted the relationship between love and death. Yes, love is what allows us to live, but it is also what allows us to face the passage of death. Without love, death is unbearable. The project has therefore enhanced love and this aspect must be highlighted. I believe that the students understood, first and foremost, that those who do not love are already dead, while those who love do not die. This is the synthesis of what we all understood together.”

## 4. Discussion

The main objectives of this study were to evaluate the efficacy of a death education initiative and its impact on teachers and hospice professionals with respect to their relationship with representations of death, and to explore how the relationship between a community and its local hospice changed following a death education initiative based on an analysis of the themes of death, dying and suicide. Our analysis of the interviews revealed three main themes and confirmed that the death education initiative resulted in meaningful changes and had a widely positive effect according to both teachers and the hospice staff. The first major theme involved changes in the way death is represented, attitudes towards life, and perspectives concerning the hospice and palliative care. This initiative modified the participants’ perspectives on end-of-life professionals, their work, and the structures in which palliative care is implemented. It also emphasised that the last moments of life can be a time of serenity, wherein the patient’s dignity is of paramount importance. The process of reconstructing the meaning of dying allowed teachers to reflect upon the valorisation of life with their students [35,38]. The teachers’ and hospice staff’s perspectives on death education confirmed the findings in the existing literature. According to various studies, death education is an important component of school curriculums because it promotes positive attitudes towards life and reflection upon existential themes [28,50]. The initiative succeeded in eliminating various barriers and cultural prejudices, thereby promoting a shift in perspective with regard to the hospice and palliative care [51].

According to the teachers, the death education experience in the hospice facilitated the elaboration of grief following a student’s suicide. Many of the teachers stressed that the opportunity to talk about death and death-related themes encouraged participants (both teachers and students) to reflect upon the value of life and the importance of respecting its fragility. Suicidal behaviours may reflect young people’s fascination with death, which may itself arise from the belief that death will free them from anxiety [52]. The scientific literature supports the idea that a more mature concept of death may prevent risky behaviours by encouraging individuals to value life [22,53]. Death education initiatives, which aim to increase social support, optimism and spirituality through specific and adaptive coping strategies can promote post-traumatic growth among those who have dealt with sudden grief [54,55,56,57]. Both the teachers and the hospice staff stressed that the death education project served as a bridge to community-wide education. Furthermore, they expressed a desire to repeat the initiative in the future. The hospice professionals confirmed that the education initiative motivated them to improve their knowledge and competencies in their everyday work, much in the way that other healthcare professionals undergo continual training to improve their relational competencies [35,58,59]. Indeed, they stated that this initiative allowed them to improve both their teamwork skills and their knowledge of themes related to death education. It also enabled them to cultivate a more positive attitude towards their work as described in previous literature [60,61,62,63]. Based on these findings, we believe that the implementation of similar death education initiatives in high schools is worthwhile.

The teachers reported that they appreciated the opportunity to visit the hospice and to have discussions with palliative care experts. They considered these encounters to be very meaningful experiences. The professionals working in this specific healthcare field are able to provide a unique point of view on the topic of death, but they were not regarded as omniscient or omnipotent, rather, the teachers and students perceived them as ordinary human beings, thus dispelling the aura of mystery and terror linked to death-related themes [40,63,64,65,66,67]. However, based on TMT and their DTA hypothesis, it is also possible that the language and coping skills learned through this program might serve to strengthen the aura of mystery surrounding death. Despite the fact that this study confirms the positive effects of death education reported in other research, wherein death education seems to promote comfort with and acceptance of thoughts about death, death education can also give people different means to actively deny death, that is, by promoting the formation of a different set of skills to proximally defend themselves against the awareness of death [68]. This issue should be carefully considered, and any elements of the program that could facilitate such defences against the awareness of death should be adjusted. In this sense, concepts from TMT, and in particular, the conceptual relationship between literal (beliefs in afterlife and in immortality of soul) and symbolic (beliefs to be an entity that transcends death) immortality should be explicitly addressed as part of death education [69,70,71,72]. Furthermore, teaching participants to recognise their own reactions to death-related thoughts could make the positive effects of death education more enduring.

## 5. Conclusions

This study confirmed the utility of creating continuity between the hospice and the community through a school-based death education program. Indeed, participants confirmed the results of our analysis, so it was possible to affirm the efficacy of the death education initiative. This last phase of the study revealed meaningful, positive changes in teachers’ approach to representations of death and to life, as well as their perceptions of end-of-life care. Hospice staff also perceived a positive change in the community’s attitudes towards them. The initiative yielded many positive outcomes. In particular, the collaboration between the hospice and high schools created a psycho-educational space in which students could explore their fears and worries regarding death while teachers could get to know their students and acquire tools to continue these discussions in the future. This space allowed the participants to elaborate their grief relating to a student’s suicide, consider death without dread, explore emotions that are usually concealed, and thus better support each other. The death education experience improved the moral environment of the schools and contributed to community building.

## 6. Limitations of the Study

One of the main limitations of the present study was that it did not involve the students who took part in the original project. In future studies, a higher number of follow-up interviews should be conducted with death education participants to assess the efficacy of such programs over time and to expand the existing literature on death education interventions for adolescents.

Furthermore, though the retrospective self-reports about the initiative were generally favourable, we did not consider whether or not people’s behaviours and current attitudes are consistent with these evaluations over time. Further research should assess the effects before, during and after the intervention, and again after a long period of time has elapsed, to see if and how previous attitudes resurface.

## Figures and Tables

**Table 1:** Participants.

	Age		Sex		Qualification	
	μ	σ	Female	Male	High School Diploma	University Degree
School (*n* = 9)	53.11	10.2	6	3	-	9
Hospice (*n* = 11)	44	5.98	6	5	4	7
Total (*n* = 20)	48.1	9.18	12	8	4	16
